# Allogeneic mesenchymal stromal cell therapy in kidney transplantation: should repeated human leukocyte antigen mismatches be avoided?

**DOI:** 10.3389/fgene.2024.1436194

**Published:** 2024-09-27

**Authors:** Suzanne Bezstarosti, Pauline Erpicum, Gianni Maggipinto, Geertje J. Dreyer, Marlies E. J. Reinders, Soufian Meziyerh, Dave L. Roelen, Johan W. De Fijter, Jesper Kers, Laurent Weekers, Yves Beguin, François Jouret, Sebastiaan Heidt

**Affiliations:** ^1^ Department of Immunology, Leiden University Medical Center, Leiden, Netherlands; ^2^ Department of Internal Medicine (Nephrology), Leiden University Medical Center, Leiden, Netherlands; ^3^ Laboratory of Translational Research in Nephrology (LTRN), Interdisciplinary Cluster for Applied Genoproteomics (GIGA) - Cardiovascular Sciences, University of Liège, Liège, Belgium; ^4^ Division of Nephrology, CHU Liège, University of Liège, Liège, Belgium; ^5^ Division of Immuno-Hematology, CHU Liège, University of Liège, Liège, Belgium; ^6^ Department of Pathology, Leiden University Medical Center, Leiden, Netherlands; ^7^ Division of Hematology, CHU Liège, University of Liège, Liège, Belgium

**Keywords:** mesenchymal stromal cells, human leukocyte antigen, kidney transplantation, epitope, eplet, donor-specific antibodies, amino acid, mismatch

## Abstract

Mesenchymal stromal cells (MSCs) have immunomodulatory properties and are therefore considered promising tools in kidney transplantation. Although most studies have been conducted with autologous MSCs, using allogeneic MSCs as an off-the-shelf product is more feasible in clinical settings. However, allogeneic MSCs could potentially induce an immune response, which might eventually be directed towards the kidney allograft because of shared human leukocyte antigen (HLA) epitope mismatches between the kidney and MSC donor. In this study, we performed in-depth analyses of two cohorts (n = 20) that received third-party MSC therapy after kidney transplantation. While the Neptune Study from Leiden University Medical Center specifically selected MSC to avoid repeated HLA antigen mismatches between kidney and MSC donors, the study from the University of Liège did not perform specific MSC selection. The comparative analyses of amino acid mismatches between these cohorts showed that MSC selection to avoid repeated HLA mismatches at the split antigen level was not sufficient to prevent repeated mismatches at the amino acid level. However, repeated amino acid mismatches were not associated with the occurrence of donor-specific antibodies (DSAs). Thus, the clinical relevance of repeated amino acid mismatches seems to be limited with regard to the risk of DSA formation. Since DSA formation was limited (3 of 20 patients) in this study, larger studies are required to investigate the relevance of preventing repeated HLA mismatches in allogeneic MSC therapy in kidney transplantation.

## 1 Introduction

Kidney transplantation is the best treatment option for patients with end-stage renal disease. Although the short-term outcomes of kidney transplantation have improved remarkably owing to the use of immunosuppressive drugs, long-term patient and graft survival have not advanced similarly mainly because of the adverse effects of chronic immunosuppression (Hariharan et al., 2021; Coemans et al., 2022). To improve graft survival, novel treatment strategies based on cellular therapy have gained interest. Mesenchymal stromal cells (MSCs) are fibroblast-like multipotent cells with anti-inflammatory, immune-regulatory, and regenerative properties, which makes them promising tools in solid organ transplantation (Vandermeulen et al., 2020). MSCs are the most studied form of cellular therapy in organ transplantation thus far, and several studies have demonstrated their safety and feasibility (Hoogduijn et al., 2021). Although the majority of studies on MSC therapy in clinical kidney transplantation have used autologous MSCs (Perico et al., 2013; Perico et al., 2018; Tan et al., 2012; Mudrabettu et al., 2015; Reinders et al., 2013), allogeneic MSCs could be more feasible sources in actual applications as these are available as an off-the-shelf product. However, the use of unmatched allogeneic MSCs could induce immune responses that may be directed against not only the MSC donor but also the kidney allograft. To the best of our knowledge, five reported studies have investigated the safety and feasibility of allogeneic MSC therapy in kidney transplantation (Sun et al., 2018; Erpicum et al., 2019; Dreyer et al., 2020; Wei et al., 2021; Ban et al., 2021), of which two have evaluated the alloimmune responses directed toward MSCs: the University of Liège (Belgium) study and the Neptune study from the Leiden University Medical Center (LUMC, the Netherlands) (Erpicum et al., 2019; Dreyer et al., 2020).

The Liège study aimed to evaluate the safety of a single infusion of third-party bone-marrow-derived MSCs in ten kidney transplant recipients under standard immunosuppression on day 3 after successful kidney transplantation; the results demonstrated that the MSCs were well tolerated and were associated with similar estimated glomerular filtration rate (eGFR) values at the end of one year as the controls. Although four of the patients developed anti-HLA antibodies against MSCs or shared kidney–MSC HLA (with only one patient showing mean fluorescence intensity (MFI) >1500), their kidney function remained stable during the 1-year follow-up, leaving the clinical relevance of these donor-specific antibodies (DSA) on the longer term unclear.

**FIGURE 1:**
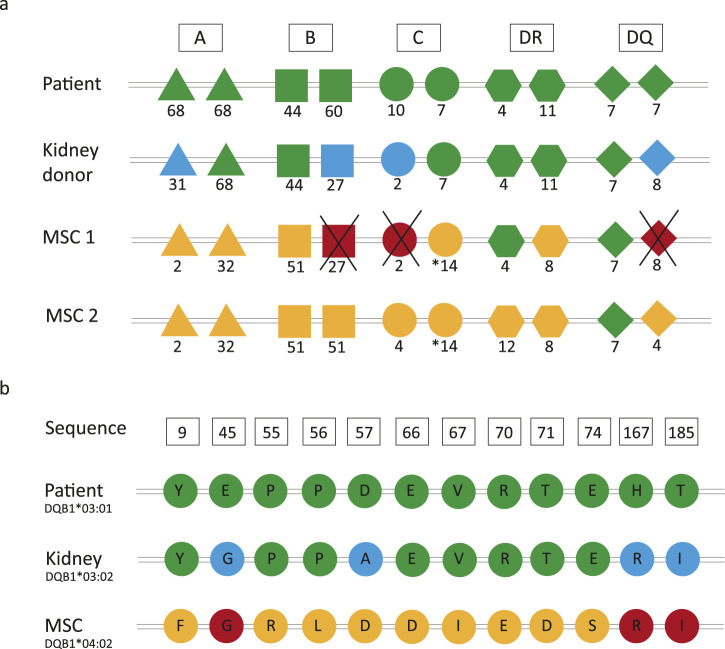
Principles of repeated human leukocyte antigen (HLA) and amino acid mismatches. **(A)** In the Neptune study, the mesenchymal stromal cell (MSC) donors were selected such that HLA mismatches occurring at the split antigen level between the recipient and kidney donor were not present between the recipient and MSC donor. **(B)** Example sequence from a patient who does not have a repeated HLA-DQB1 antigen mismatch (patient: DQ7, kidney: DQ8, and MSC: DQ4) but still has three repeated amino acid mismatches. Color legend: green, typing of the recipient; blue, mismatches between the recipient and kidney donor; yellow, mismatches between the recipient and MSC donor; red, repeated mismatches.

The Neptune study from the LUMC also involved the use of bone-marrow-derived third-party MSC donors that were administered 6 months after transplantation. The primary aim of this study was to prove the safety and feasibility of infusing third-party MSCs. In order to prevent that a DSA directed towards the MSC donor could possibly also be directed against the kidney allograft, MSC donors were selected to prevent repeated HLA mismatches with the kidney donor at the split antigen level. This means that the MSC donor could not have any HLA antigen mismatches with the recipient that were already present on the kidney donor (Figure 1A). The Neptune study demonstrated that MSC treatment was feasible and safe and that no *de novo* DSAs were found one year after transplantation.

DSAs are induced by mismatched polymorphic amino acids on the HLA molecules of the donor. Although each HLA allele has its own unique amino acid sequence, polymorphic amino acids may be shared between different HLA molecules (Tambur and Claas, 2015; Bezstarosti et al., 2022). Multiple studies have demonstrated that amino acid mismatches are associated with inferior transplant outcomes, and an increasing body of evidence suggests that HLA immunogenicity analysis should be performed on the amino acid instead of the antigen levels (Kosmoliaptsis et al., 2016; Wiebe et al., 2018; Delion et al., 2019; Kramer et al., 2020; Meneghini et al., 2020). Consequently, this means that although there may not be repeated mismatches at the HLA antigen level, there could still be repeated mismatches at the amino acid level. This is illustrated in Figure 1B, which shows that even if there is no repeated antigen mismatch between the kidney and the MSC donor, there are still three repeated amino acid mismatches that could potentially induce DSA formation.

The use of different strategies for MSC donor selection in the setting of kidney transplantation raises the question of whether it is necessary to avoid repeated HLA mismatches between the kidney and MSC donors at the antigen or amino acid level to prevent DSA formation against the shared mismatches. With the aim of providing additional insights into the preferred selection method of allogeneic MSCs for cellular therapy in kidney transplantation, we determined the number of repeated amino acid mismatches in the Liège as well as Neptune MSC study cohorts and investigated whether these repeated amino acid mismatches were associated with DSA formation.

## 2 Methods

### 2.1 Description of the study cohorts

The design characteristics of the MSC study at the University of Liège and Neptune study at the LUMC are depicted in Table 1 (Erpicum et al., 2019; Dreyer et al., 2020). In brief, the patients enrolled in the Liège study received transplants from deceased donors (donation after brain or circulatory death), while those in the Neptune study received living donor transplants (related or unrelated). Both studies were monocentric and included ten first-time kidney transplant recipients to receive bone-marrow-derived MSCs from third-party donors. The Liège patients received one infusion of MSCs on day 3 post-transplantation, while the Neptune patients received two infusions each at week 25 and 26 post-transplantation. In the Neptune study, the MSC donors were selected such that there were no repeated HLA mismatches at the split antigen level for HLA-A, -B, -DR, and -DQ. This is in contrast with the Liège study cohort, where the patients were treated with third-party MSCs without specific HLA selection. The induction and maintenance of immune suppression were different in both cohorts. The baseline characteristics of the included patients have been described previously (Erpicum et al., 2019; Dreyer et al., 2020) and are summarized in Table 2.

**TABLE 1:** Study characteristics of the two clinical trials.

Characteristic	Liège study^a^	Neptune study Leiden^b^
Phase	Phase I-II single center	Phase Ib single center
EUDRA CT	2011-001822-81	2013-005407-14
ClinicalTrials.gov identifier	NCT01429038	NCT02387151
Number of patients	10	10
Duration of the primary study	12 months	12 months
Population	18–75 years, fist KTx, PRA ≤50%	18–75 years old first KTx recipients, PRA ≤50%
Kidney donor	Deceased (DBD or DCD)	Living (related or unrelated)
Time of MSC infusion	One infusion at day 3 ± 2 post-Tx	Two infusions at weeks 25 and 26 post-Tx
Dosage of MSCs	1.5 × 10^6^ to 3 × 10^6^/kg bodyweight	1.5 × 10^6^/kg bodyweight
Source of MSCs	Bone marrow, third-party donors	Bone marrow, third-party donors
Selection of the MSC donor	No HLA selection	Avoidance of repeated HLA-A, B, -DR, and -DQ antigen mismatches
Induction for MSCs	Methylprednisolone at 2 mg/kg bodyweight and promethazine	Not performed
Induction for KTx	Anti-interleukin-2 receptor antibodies on day 0 and day 4	Alemtuzumab
Maintenance immunosuppression	Tacrolimus, mycophenolate mofetil, and corticosteroids	Prednisone, tacrolimus, and everolimus
Immunosuppression minimization	Not applicable	Reduction of tacrolimus to trough levels of 1.5–3 ng/mL after the second MSC infusion
Primary endpoint	Adverse effects of MSC infusion as well as infectious and malignant complications at 1 year	Biopsy-proven acute rejection or graft loss

^a^ Erpicum et al. Kidney Int. 2019 March; 95 (3):693-707.

^b^ Dreyer et al. Am J Transplant. 2020 October; 20 (10):2905-2915.

DBD, donor after brain death; DCD, donor after circulatory death; KTx, kidney transplantation; MSC, mesenchymal stromal cell; PRA, panel-reactive antibody.

**TABLE 2:** Baseline characteristics of the participants.

Characteristic	Liège study (N = 10)	Neptune study Leiden (N = 10)
Recipients		
Age at transplantation (year)	63 (54–67)	52 (43–59)
Sex: male/female	7/3	6/4
CDC PRA max <5%/5%–84%/>85%	9/1/0	7/3/0
Kidney donors		
Age (year)	52 (47–57)	60 (49–63)
Sex: male/female	3/7	4/6
Transplantation		
DBD/DCD/living	7/3/0	0/0/10
CMV status 1: D+/R+; 2: D+/R-; 3: D-/R+; 4: D-/R-	3/3/3/1	2/0/2/6
Cold ischemia time (min)	716 (504–814)	188 (172–200)
Warm ischemia time (min)	45 (40–57)	27 (26–33)

CDC PRA, complement-dependent cytotoxicity panel-reactive antibody; CMV, cytomegalovirus; D, donor status; DBD, donor after brain death; DCD, donor after circulatory death; R, recipient status.

Data are expressed as median value (P25–P75) for continuous variables and as number for categorical variables.

### 2.2 HLA typing and repeated mismatch analysis

For the purpose of this study, the recipients, kidney donors, and MSC donors were retrospectively HLA typed for 11 loci at high resolution (minimum second-field level) using next-generation sequencing (NGS) on an Illumina platform (Illumina, San Diego, CA, United States) and NGSgo kits (GenDx, Utrecht, the Netherlands).

Repeated HLA mismatches were assessed at the split antigen and amino acid levels (Figure 1). A repeated mismatch was defined as a mismatch between the recipient and kidney donor that is also present between the recipient and MSC donor. The HLA epitope mismatch algorithm (HLA-EMMA) version 1.06 (https://hla-emma.com/) was used to define HLA class I (intralocus) as well as HLA-DR and HLA-DQ (interlocus) solvent-accessible amino acid mismatches between the recipient and kidney donor and between the recipient and MSC donor (Kramer et al., 2020). Recipient/kidney donor mismatches were compared with recipient/MSC donor mismatches to identify repeated mismatches.

### 2.3 Donor-specific antibody detection

In the Liège study, sera were tested using the Lifecodes single-antigen bead (SAB) assay (Immucor) at 1, 3, 6, and 12 months after transplantation (Erpicum et al., 2019). After the 1-year study period, the patients were annually screened for DSAs using SAB during the routine outpatient clinic follow-up. In the Neptune study, sera were screened for anti-HLA antibodies at baseline as well as weeks 24, 30, and 52 after transplantation (Dreyer et al., 2020). All samples from weeks 24 and 52 were additionally tested with the Lifecodes SAB kit. After the initial 1-year study period, screening was performed at the time of graft dysfunction or annually depending on serum availability using the LABScreen kit (One Lambda) or Lifecodes LifeScreen Deluxe kit (Immucor). In the case of positive screening, the HLA antibody specificity was assessed using the Lifecodes SAB kit. Data were analyzed using Match It! Antibody software (Immucor). A background-corrected mean fluorescence intensity (BCM) ≥ 1,000 was considered positive.

### 2.4 Clinical follow-up

The patients in both cohorts were followed-up annually at the outpatient clinic after the initial 1-year study period. The data on kidney function (eGFR calculated through CKD-EPI and proteinuria), graft survival, and patient survival were collected until 5 years of follow-up.

### 2.5 Statistical analysis

Data analysis was performed using IBM SPSS Statistics version 25 (IBM Corp., Armonk, NY, United States). Categorical variables were summarized using counts, and continuous variables were described in terms of the median and interquartile range or mean ± standard deviation. A *p*-value <0.05 was considered to be statistically significant. Patients who experienced graft loss, which is defined as return to dialysis or retransplantation, were included with eGFRs of 10 mL/min/1.73 m^2^ from graft loss onwards. Finally, univariate comparisons were performed using the Mann–Whitney test.

## 3 Results

### 3.1 Donor-specific antibody formation

**FIGURE 2:**
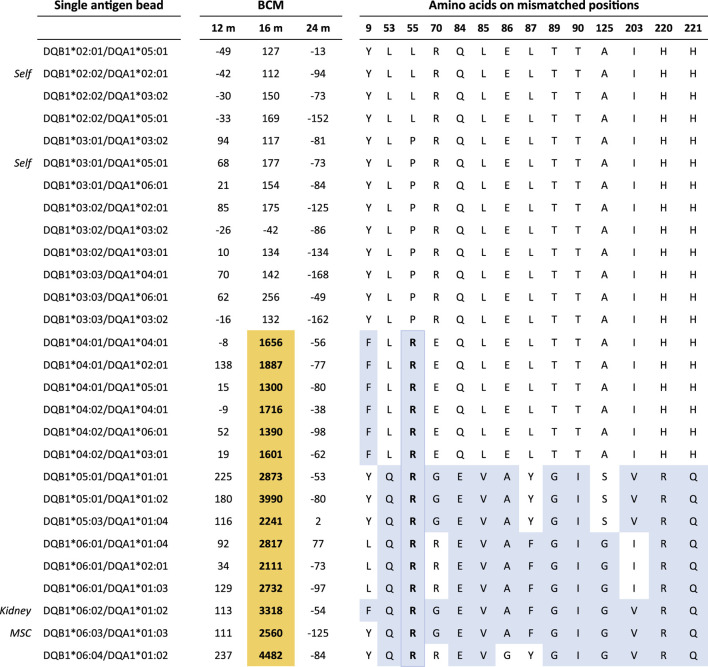
Single-antigen bead data on Liège patient #1 at 12, 16, and 24 months post-transplantation. Residue 55R is uniquely shared by all reactive beads at month 16. Color legend: orange, positive beads; blue, amino acid mismatches between the recipient and kidney/MSC donors. BCM, background-corrected mean fluorescence intensity.

As reported previously, four patients in the Liège cohort developed DSAs in the first year after transplantation (Erpicum et al., 2019). Since high-resolution typing was not available at the time of the initial DSA analysis, we reanalyzed the raw SAB data in light of the newly acquired high-resolution HLA typing data. For patient #1, the anti-DQ6 DSA directed against the MSC donor were described at 1 month after transplantation; in this case, the imputed high-resolution HLA-DQ typing was DQB1*06:02 for the kidney donor and DQB1*06:04 for the MSC donor, while the patient was DQ2 and DQ7. The previous DSA classification was based on the positivity of the DQB1*06:04/DQA1*01:01 bead in the SAB assay, while the other DQB1*06 beads were negative. However, high-resolution typing showed that the typing of the MSC donor actually was DQB1*06:03. Therefore, the reactivity against the DQB1*06:04/DQA1*01:01 bead is likely to be an artifact of the SAB assay. Analysis of the SAB data beyond the first year of transplantation showed a DSA at 16 months against DQB1*06:02 (kidney donor) with a BCM of 3,318. The reactivity pattern of the antibodies in the SAB assay (Figure 2) suggests that this DSA is directed against the amino acid 55R, which is a repeated amino acid mismatch as it is also present on the mismatched HLA of the MSC donor (DQB1*06:03). Indeed, the DQB1*06:03-carrying bead was also positive with a BCM of 2,560. No DSA were detected at years 2, 3, 4, and 5.

For patient #4 in the Liège cohort, antibodies against HLA-B*51:01 of the kidney donor were detected 6 months after transplantation (BCM: 1,342). At the same time, antibodies against A*26:01 (BCM: 1,002) and C*06:02 (BCM: 1,897) of the MSC donor were found. However, the self-allele B*44:02 had a similar MFI value (BCM: 1,364). Therefore, we considered the reactivity in this SAB assay as background and not as DSAs. No DSA were detected at 3, 4, and 5 years after transplantation.

Lastly, two more patients from the Liège cohort (#3 and #8) showed DSAs within 1 year of transplantation. Unfortunately, no DNA material was available for high-resolution typing, so it was not possible to perform repeated mismatch analysis on the amino acid mismatch level. We chose not to perform imputation of high-resolution HLA typing for these two cases as previous research has shown that this can lead to incorrect DSA assignment at the individual patient level (Senev et al., 2020). Considering the HLA typing at the serologic split level, the DSA of patient #3 were directed against DQ2, which was shared between the kidney and MSC donor. This means that it could theoretically have been induced by a shared epitope. However, this cannot be confirmed without high-resolution typing of the recipient, kidney donor, and MSC donor as DQ2 can correspond to either HLA-DQB1*02:01 or DQB1*02:02. These two alleles have distinct amino acid mismatches between them, which means that if there is an allele mismatch between the kidney and MSC donor, it is possible that the DQ2-directed DSA would not be directed against a repeated amino acid mismatch.

Another patient whose high-resolution typing data were not available is patient #8; this patient developed DSAs against DQ5, which was a mismatch with the MSC donor but not with the kidney donor. Theoretically, these DSAs could have been induced by the MSC donor. However, without high-resolution HLA typing, it cannot be confirmed that the DSAs are indeed directed against the HLA of the MSC donor, because DSA assignment based on serological data alone can lead to DSA misclassification, as shown for patient #1. Furthermore, based on serological typing, there were no HLA-DQ mismatches between the recipient and kidney donor. However, without high-resolution typing data, an allele mismatch between the recipient and kidney donor cannot be excluded. Hence, based on first-field HLA typing alone, it is not possible to attribute the detected DSAs to the MSC or kidney donor.

In the Neptune cohort, a total of 85 serum samples were screened using Luminex. As reported previously, the per-protocol SAB tests of the serum samples at weeks 24 and 52 showed no DSAs. No DSAs were identified during the additional follow-up.

### 3.2 Repeated HLA amino acid mismatch analysis

Two of the ten Liège patients were not included in the repeated amino acid mismatch analysis because of a lack of sufficient material for high-resolution typing. A total of nine repeated antigen mismatches were observed in four out of the remaining eight patients. In the other four patients, although there was no HLA selection, there were also no repeated antigen mismatches (Table 3). The median values of the HLA class I, HLA-DR, and HLA-DQB1 repeated amino acid mismatches were 4.5, 4.0, and 5.5 respectively.

**TABLE 3:** Repeated mismatch analyses of the Liège study patients.

		Antigen split mismatch					Amino acid mismatch		
		A	B	C	DR	DQ	Class I	DR	DQB
L1	Kidney	2	1	2	1	1	12	9	14
	MSC	1	0	1	1	1	12	1	13
	Repeated	0	0	0	0	1	4	0	13
L2	Kidney	1	0	1	1	1	10	4	21
	MSC	0	1	1	0	1	5	0	12
	Repeated	0	0	0	0	0	2	0	8
L3^a^	Kidney	1	1	0	0	1			
	MSC	1	1	1	1	2			
	Repeated	1	1	0	0	1			
L4	Kidney	1	1	1	1	0	7	15	0
	MSC	2	2	2	2	2	18	26	15
	Repeated	0	0	0	0	0	6	15	0
L5	Kidney	1	1	1	1	1	15	16	9
	MSC	2	1	1	1	1	18	3	8
	Repeated	1	0	0	0	0	11	3	1
L6	Kidney	1	2	2	1	1	18	16	2
	MSC	2	2	2	2	2	18	21	10
	Repeated	1	1	1	1	1	13	16	2
L7	Kidney	1	1	1	1	1	17	15	23
	MSC	1	1	2	2	2	12	29	28
	Repeated	0	0	0	1	1	5	15	23
L8*	Kidney	2	2	2	0	0			
	MSC	2	2	1	2	2			
	Repeated	0	0	0	0	0			
L9	Kidney	2	1	2	1	1	7	4	16
	MSC	2	2	2	2	1	10	36	22
	Repeated	0	0	0	0	0	0	3	11
L10	Kidney	0	0	2	1	1	5	11	12
	MSC	2	1	1	1	1	15	17	16
	Repeated	0	0	0	0	0	1	5	3

^a^ No material was available for high-resolution HLA typing, so amino acid mismatches were not analyzed.

**FIGURE 3:**
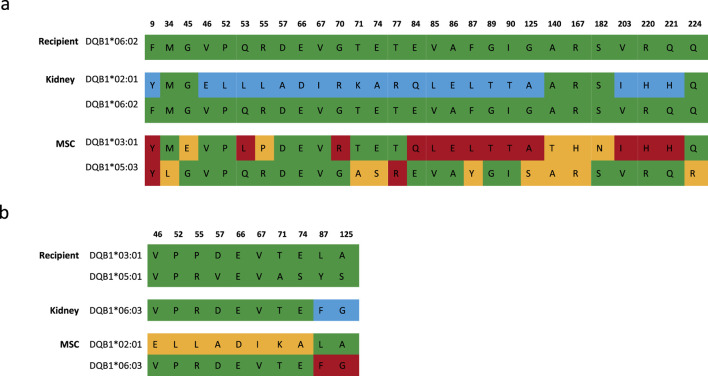
Solvent-accessible amino acid mismatches of DQB1 between the recipient, kidney donor, and MSC donor. **(A)** DQB1 typing of Neptune patient #10 showed no repeated antigen mismatches but 14 repeated amino acid mismatches. **(B)** DQB1 typing of Liège patient #6 showed a single repeated antigen mismatch (DQ6) and two corresponding repeated amino acid mismatches. Color legend: green, typing of the recipient; blue, mismatches between the recipient and kidney donor; yellow, mismatches between the recipient and MSC donor; red, repeated mismatches. MSC, mesenchymal stromal cell.

Despite the fact that MSCs were specifically selected in the Neptune cohort to avoid repeated antigen mismatches at the HLA-A, -B, -DR, and -DQ levels, one repeated mismatch occurred at HLA-DQ because a suitable MSC product could not be found and this repeated mismatch was accepted. Although there was no selection for HLA-C, only one repeated mismatch was found at the HLA-C level (Table 4). The median values of the HLA class I, HLA-DR, and HLA-DQB1 repeated amino acid mismatches were 3.0, 0.5, and 2.5 respectively. Interestingly, although patient #10 did not have any repeated antigen mismatches for HLA-DQB1, there were a large number of repeated amino acid mismatches: 14. This was caused by the shared amino acids between DQ2 (kidney mismatch) and DQ7 (MSC mismatch), which emphasizes that selection at the HLA level does not imply zero or a low level of repeated mismatches at the amino acid level (Figure 3A). In contrast, patient #6 from the Liège cohort had a repeated HLA-DQ antigen mismatch but only two repeated amino acid mismatches (Figure 3B).

**TABLE 4:** Repeated mismatch analyses of the Neptune study patients.

		Antigen split mismatch				Amino acid mismatch			
		A	B	C	DR	DQ	Class I	DR	DQB
N1	Kidney	1	1	1	0	1	11	0	4
	MSC	2	1	2	2	1	12	8	11
	Repeated	0	0	0	0	0	5	0	3
N2	Kidney	2	2	1	2	2	12	22	26
	MSC	0	1	2	2	2	11	31	19
	Repeated	0	0	0	0	1^a^	3	8	18
N3	Kidney	1	2	1	1	1	8	25	8
	MSC	1	2	1	0	0	14	0	0
	Repeated	0	0	0	0	0	5	0	0
N4	Kidney	0	1	1	1	1	4	4	18
	MSC	1	2	2	1	1	10	11	5
	Repeated	0	0	1	0	0	3	0	2
N5	Kidney	1	2	1	1	1	9	3	14
	MSC	1	0	1	2	1	7	5	7
	Repeated	0	0	0	0	0	3	0	2
N6	Kidney	1	2	1	0	1	15	0	1
	MSC	1	2	1	2	2	12	19	19
	Repeated	0	0	0	0	0	7	0	1
N7	Kidney	0	1	1	1	1	7	11	19
	MSC	0	1	1	2	1	3	5	7
	Repeated	0	0	0	0	0	2	1	2
N8	Kidney	2	0	2	1	1	10	24	14
	MSC	0	2	0	1	1	4	16	18
	Repeated	0	0	0	0	0	0	7	4
N9	Kidney	1	2	1	2	1	11	22	9
	MSC	0	2	2	2	2	13	29	9
	Repeated	0	0	0	0	0	4	10	3
N10	Kidney	0	0	0	1	1	0	20	22
	MSC	1	2	0	2	2	9	38	25
	Repeated	0	0	0	0	0	0	15	14

^a^ Repeated HLA-DQ antigen mismatch accepted due to lack of suitable MSC donors without repeated antigen mismatches.

The total repeated amino acid mismatches (HLA class I + HLA-DR + HLA-DQ) was higher in the Liège cohort, but this was not statistically significant (median 16 vs. 8, *p* = 0.056, Mann–Whitney test). Importantly, the likely target of the DSAs of Liège patient #1, namely eplet 55R, was also repeatedly mismatched in one more Liège patient and in two Neptune patients. However, none of these patients developed DSAs against this target.

### 3.3 Clinical follow-up

**FIGURE 4:**
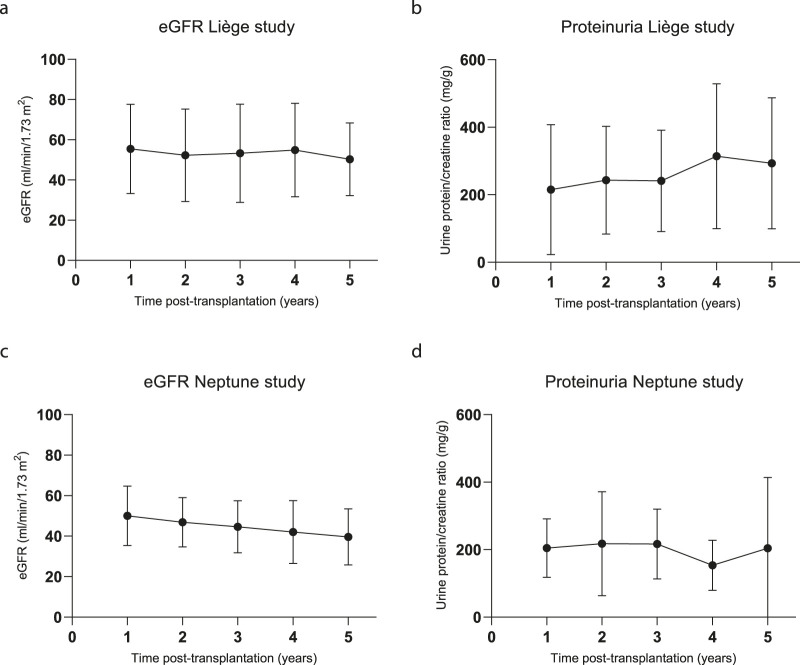
Kidney function during the 5-year follow-up. **(A)** Estimated glomerular filtration rate (eGFR in mL/min/1.73 m^2^) calculated using the CKD-EPI formula for the Liège study patients; N = 10 for all time points. **(B)** Urine protein/creatinine (mg/g) ratios of the Liège study patients; n = 9, 8, 8, 7, and 8 for years 1, 2, 3, 4, and 5, respectively. **(C)** eGFR calculated using the CKD-EPI formula and **(D)** urine protein/creatinine ratios of the Neptune patients; n = 10, 10, 10, 9, and 8 for years 1, 2, 3, 4, and 5, respectively.

The kidney function results (eGFR and protein/creatinine ratio) of the patients in both cohorts over the course of 5 years are depicted in Figure 4. As per the 5-year follow-up findings, Liège patient #5 experienced graft failure at year 3 after transplantation, returned to dialysis, and died from a massive cerebral hemorrhage at 5 years post-transplantation; in this patient, borderline rejection was identified from the surveillance biopsy at 3 months post-transplantation (Erpicum et al., 2019), but no DSAs were detected. A for-cause biopsy at month 8 showed BK nephropathy. Three other patients underwent for-cause biopsies during the 5-year follow-up, from which T-cell-mediated rejection (Banff IB, day 330, patient #2, no DSAs) and borderline rejection (day 233, patient #3) were diagnosed. Importantly, the for-cause biopsy performed on patient #1 at the time of DSA detection (16 months post-transplantation) did not show any signs of rejection. Five years after transplantation, patient survival was 100% in the Neptune study, and none of the patients experienced graft failure. One patient underwent for-cause biopsy (day 527) and showed chronic-active T-cell-mediated rejection (Banff IA), which was successfully treated with methylprednisolone.

## 4 Discussion

Although most MSC studies have been conducted with autologous MSCs, allogeneic MSCs could be a more feasible treatment as an off-the-shelf product. Since there is limited data on the immunogenicity of allogeneic MSCs, we performed an in-depth analysis of two patient cohorts that received third-party MSC therapy after kidney transplantation. The first cohort consisted of 10 patients from the University of Liège, Belgium, who received MSC therapy on day 3 after kidney transplantation (Erpicum et al., 2019). Since high-resolution typing had not been performed initially, all recipients and donors were typed for 11 HLA loci, and their SAB data were reanalyzed. In the original study, the single antigen bead assay was considered positive if two of the following three criteria were met: BCM > 1500, BCR > 5, or AD-BCR > 5. In the current study, a cutoff of BCM > 1000 was used. The reanalysis of the SAB data in light of the high-resolution typing data offered several different conclusions than the initial analyses. First, the anti-DQB1*06:04 DSAs against the MSC donor of patient #1 that were detected at 1 month post-transplantation appeared to be misclassified since the actual typing of the MSC donor was DQB1*06:03. Indeed, Senev et al. (2020) demonstrated that inferring high-resolution haplotypes based on low-resolution typing led to misclassification of almost 25% of the DSAs. The SAB data beyond the first year after transplantation showed a broader reactivity pattern, in which DQ4, DQ5, and DQ6, including the kidney donor allele DQB1*06:02 and MSC donor allele DQB1*06:03, were positive. Amino acid mismatch analyses showed that the amino acid 55R corresponding to eplet 55R (Bezstarosti et al., 2021a; Bezstarosti et al., 2021b) was uniquely shared by the reactive alleles and was not present on the HLA of the recipient. Residue 55R is a repeated amino acid mismatch as it is also present on the DQB1*06:03 allele of the MSC donor. However, it is unlikely that the DSAs that were detected at 16 months after transplantation were elicited by the MSC donor, as the MSCs were administered several days after transplantation and have been shown to be short-lived as well as become trapped in venous capillaries of the lungs (Eggenhofer et al., 2012; Barbash et al., 2003).

The importance of second-field high-resolution typing for accurate DSA assignment was illustrated in Liège patient #4. In this case, the patient’s initial HLA-B low-resolution typing was B44 and B49, while there was a B51 mismatch with the kidney donor. Based on the reactivity of the B*51:01 bead in the SAB assay, anti-B51 DSAs were assigned. However, reanalysis of the SAB data combined with the second-field typing of the patient showed that the self-allele B*44:02 had a similar reactivity as bead B*51:01. In SAB assays with such high background signals against self HLA alleles, it is problematic to assign DSAs; therefore, we chose not to consider this as a DSA.

In contrast to the Liège cohort, there was no DSA formation in the Neptune cohort from the LUMC (Dreyer et al., 2020). While there was no HLA-based selection of the MSC donors in the Liège study, the Neptune study protocol aimed to avoid repeated HLA mismatches between the kidney and MSC donors at the antigen split level. Hence, we performed amino acid mismatch analysis to determine whether the MSC selection procedure in the Neptune study would lead to fewer repeated amino acid mismatches and whether this strategy would be associated with lower DSA formation. Our results demonstrate that patients in both cohorts display a wide range of amino acid mismatches between recipient and donors, among which several are shared mismatches between the kidney and MSC donor. Although we showed that the Neptune patients had fewer repeated amino acid mismatches on average, the individual patient cases demonstrate that the absence of repeated HLA mismatches does not automatically mean the absence of repeated amino acid mismatches. Conversely, the presence of a repeated antigen mismatch does not necessarily mean that there is a high number of repeated amino acid mismatches, as illustrated in Figure 3. Most importantly, the number of repeated amino acid mismatches does not seem to be associated with DSA formation. Furthermore, in patients who developed DSAs, the DSAs were most likely induced by mismatches with the kidney donor and not by the MSC donor. Therefore, the differences in DSA formation between the two cohorts are likely influenced by differences in the transplantation type (deceased versus living), (Coemans et al., 2022), immunosuppression, and (Vandermeulen et al., 2020) the MSC infusion and culture protocols. The timing of the MSC infusion (i.e., immediately or several months after kidney transplantation) may also influence the immunogenicity of the MSCs as preclinical studies have shown microenvironment-driven changes in the MSC expression of major histocompatibility complex (MHC) class II (Gu et al., 2015).

Although MSCs were initially considered to be immune-privileged, *in vitro* and *in vivo* models have demonstrated that allogeneic MSCs can induce both cellular and humoral immune responses and can be rejected (Ankrum et al., 2014; Berglund et al., 2017). While data on sensitization by MSCs are limited, clinical trials investigating allogeneic MSC therapies in cardiomyopathy, osteoarthrosis, and Crohn’s disease did not report significant antibody formation against MSCs (Hare et al., 2012; Ascheim et al., 2014; García-Sancho et al., 2017; Hare et al., 2017; Avivar-Valderas et al., 2019). In a recent study, liver transplant patients treated with allogeneic MSCs developed more DSAs than the controls; in total, six patients developed DSAs, among whom the DSAs of three patients were directed against a shared mismatch between the liver allograft and MSCs. Because all of the MSC DSAs were directed against a repeated mismatch with the liver donor, it was difficult to determine whether these DSAs were induced by the liver allograft or whether the MSCs induced or contributed to the immune response (Vandermeulen et al., 2022; Detry et al., 2017).

The present study is limited by the small sample size of 20 patients, of whom two had to be excluded because material for high-resolution HLA typing was lacking. Because only a small number of patients developed DSAs, the statistical power and generalizability of the results are limited. Second, the two study cohorts significantly differed in their design regarding donor types (deceased vs. living), timing of MSC infusion (day 3 vs. week 25 post-transplantation), and immunosuppressive regimen. Although all patients were first-time transplant recipients and had baseline characteristics that were relatively comparable between the two cohorts, other factors that could have influenced DSA formation, such as their underlying health conditions, cannot be excluded. Furthermore, the kits used for the DSA screening were different for the two study cohorts. Although discrepancies have been noted between the two vendors (Clerkin et al., 2017), we believe that this does not have a significant effect on the results since the serum samples were tested extensively in not only the luminex screening assay but also the SAB assay. Moreover, only three patients developed DSAs. Lastly, it is important to emphasize that the primary aim of this study was not to compare the clinical outcomes of the Neptune and Liège studies but provide a detailed analysis of the amino acid mismatches to gain insights into the preferable method for MSC product selection. The main strengths of this study include detailed analyses of the repeated amino acid mismatches between the kidney and MSC donors, 5-year follow-up time for DSA development, and SAB assays that were routinely performed in both study cohorts.

In conclusion, the present study shows that selection of MSC donors to avoid repeated HLA mismatches at the antigen split level is not sufficient to prevent repeated mismatches at the amino acid level. Therefore, if repeated HLA mismatches need to be avoided, HLA matching at the amino acid level is required. Nevertheless, the clinical relevance of preventing repeated amino acid mismatches in MSC donors to avoid the risk of DSA formation seems to be limited. Only one patient developed DSAs against a repeated mismatch, which was likely induced by the kidney donor and not the MSC donor. Other patients did not develop DSAs against repeated mismatches. As third-party allogeneic MSCs are promising tools in solid-organ transplantation, future research should include detailed analyses of the immune response against allogeneic MSCs in larger cohorts, for which second-field HLA typing is critical for comprehensive analyses.

## Data Availability

The raw data supporting the conclusions of this article will be made available by the authors without undue reservation.

## Abbreviations

BCM, background-corrected mean fluorescence intensity; DSA, donor-specific antibody; eGFR, estimated glomerular filtration rate; HLA, human leukocyte antigen; HLA-EMMA, HLA epitope mismatch algorithm; MFI, mean fluorescence intensity; MSC, mesenchymal stromal cell; NGS, next-generation sequencing; SAB, single-antigen bead.
